# Strategies for the Successful Implementation of a Novel iPhone Loaner System (iShare) in mHealth Interventions: Prospective Study

**DOI:** 10.2196/16391

**Published:** 2019-12-16

**Authors:** William E Yang, Erin M Spaulding, David Lumelsky, George Hung, Pauline Phuong Huynh, Kellen Knowles, Francoise A Marvel, Valerie Vilarino, Jane Wang, Lochan M Shah, Helen Xun, Rongzi Shan, Shannon Wongvibulsin, Seth S Martin

**Affiliations:** 1 School of Medicine Johns Hopkins University Baltimore, MD United States; 2 School of Nursing Johns Hopkins University Baltimore, MD United States; 3 Krieger School of Arts and Sciences Johns Hopkins University Baltimore, MD United States; 4 Ciccarone Center for the Prevention of Cardiovascular Disease Division of Cardiology School of Medicine, Johns Hopkins University Baltimore, MD United States; 5 Department of Biomedical Engineering School of Medicine Johns Hopkins University Baltimore, MD United States

**Keywords:** mHealth, digital health, innovation, myocardial infarction, health care disparities, smartphone, mobile phone, smart technology, loaner device, telemedicine

## Abstract

**Background:**

As smartphone ownership continues to rise, health care systems and technology companies are driven to develop mobile health (mHealth) interventions as both diagnostic and therapeutic tools. An important consideration during mHealth intervention development is how to achieve health equity despite demographic differences in smartphone ownership. One solution is through the recirculation of loaner smartphones; however, best practices for implementing such programs to optimize security, privacy, scalability, and convenience for participants are not well defined.

**Objective:**

In this tutorial, we describe how we implemented our novel Corrie iShare program, a 30-day loaner iPhone and smartwatch recirculation program, as part of a multi-center mHealth intervention to improve recovery and access to guideline-directed therapy following acute myocardial infarction.

**Methods:**

We conducted a prospective study utilizing a smartphone app and leveraged iOS enterprise features as well as cellular data service to automate recirculation.

**Results:**

Our configuration protocol was shortened from 1 hour to 10 minutes. Of 200 participants, 92 (46.0%) did not own an iPhone and would have been excluded from the study without iShare. Among iShare participants, 72% (66/92) returned their loaned smartphones.

**Conclusions:**

The Corrie iShare program demonstrates the potential for a sustainable and scalable mHealth loaner program, enabling broader population reach while optimizing user experience. Implementation may face institutional constraints and software limitations. Consideration should be given to optimizing loaner returns.

## Introduction

Mobile health (mHealth) interventions are promising therapeutic tools [1]. The high rate of smartphone ownership among Americans, up to 77% in 2018 [2], and the popularity of wearable technologies have driven health care systems and technology companies to develop mHealth interventions and create innovative health care delivery methods.

The delivery platform critically influences mHealth functionality. Google’s Android and Apple’s iOS dominate market share, accounting for 99.8% of smartphones [3]. mHealth app developers need to choose between developing a cross-platform app or native apps. Web-based (ie, *cross-platform*) apps require little additional work to reach both major smartphone platforms. Although *cross-platform* apps have fast time to market and require less maintenance, they are unable to take advantage of all the features that a *native* platform-specific app could. On the other hand, developing multiple native apps, in addition to requiring more time and resources, also becomes hindered by the lack of interoperability between platforms (eg, the Apple Watch cannot be paired with an Android phone).

Platform selection also has implications for health equity due to demographic differences in smartphone ownership. In 2013, those who identified as *black, non-Hispanic*, had less than a college education, or whose annual household income was below US $75,000 were more likely to own an Android phone than an iPhone [4]. As socioeconomic status contributes to health disparities, being inclusive of socioeconomically disadvantaged groups when choosing an mHealth intervention platform offers an opportunity to reduce health disparities.

Our team was cognizant of these factors as we developed the *Corrie* app for the Myocardial infarction COmbined-device Recovery Enhancement (MiCORE) prospective study (Johns Hopkins University: IRB00099938, NCT03760796). We considered multiple approaches to balance inclusiveness and optimal user experience. Although we initially focused on cross-platform development, Corrie was ultimately developed as a native iOS app following the release of the Apple CareKit health app framework in 2016 [5]. iOS secures and encrypts protected health information (PHI), while native app development enables smartwatch integration, interactive notifications, calendar and contacts integration, and Bluetooth.

Prior mHealth studies utilizing loaner smartphones have not described how to implement a loaner model [6-8], details of which are key to ensuring security, privacy, scalability, and sustainability. To address these details and maintain broad population reach, we created the *iShare* loaner program. Through iShare, we enrolled patients who did not own an iPhone into MiCORE by providing each with a reusable loaner iPhone and Apple Watch preloaded with Corrie. In this paper, we describe the development of our novel loaner program, initial findings, and considerations for its generalized application.

## Methods

### Toward a Sustainable and Scalable Protocol

#### Overview

Without experience or guidance regarding the best approach to operationalize a smartphone loaner program for a clinical research study, we developed the program iteratively. With each iteration came an increased financial investment, often resulting in a greater-than-anticipated improvement in our processes. Table 1 provides a summary of our protocol’s evolution. While our final protocol (see Multimedia Appendix 1) was optimized for the MiCORE study, earlier iterations may be more appropriate for other mHealth studies. Determining optimal configuration settings also would have been challenging without the experience gained during earlier iterations.

At the advent of iShare, rather than purchasing new iPhones, we chose the economical approach of purchasing refurbished iPhones in small batches from third-party resellers. Apple donated Apple Watches for the MiCORE study.

**Table 1:** Use cases and characteristics of each protocol version.

Characteristic	Version 1.0	Version 2.0	Version 3.0
Use case	Exploratory testing of platform capabilities	Small-scale study	Study requiring scalability
Interactive time	30-60 minutes Lowest level of automation	20-30 minutes	5-10 minutes Highest level of automation
Requirements	Minimum financial investment Study team Apple ID for each phone Participants must have home Wi-Fi USB connection required to reset PIN-locked phone	In addition to previous requirements: Apple Mac computer running Apple Configurator software Low-cost SIMs without cellular service	Apple Mac computer running Apple Configurator software Apple Business Manager (free) Mobile Device Management (subscription fee) Cellular plan for each loaner phone
Features	Unrestricted Apple App Store Activation Lock to prevent circumventing security measures by factory reset Find My iPhone GPS tracking to reduce the risk of theft iOS restrictions to disable iCloud and prevent inadvertent sharing of PHI^a^	Increased consistency in configuration due to automation	Apple Device Enrollment Program to prevent circumventing security measures by factory reset Lost Mode GPS tracking to reduce the risk of theft Mobile Device Management policies to disable iCloud and prevent inadvertent sharing of PHI
Limitations	Manual (error-prone) configuration of all features and app installation Insertion and removal of SIM card required to activate phones after each reset Find My iPhone not helpful outside of Wi-Fi range	Manual configuration of Apple ID and app installation Find My iPhone not helpful outside of Wi-Fi range Apple Configurator unable to share configuration profiles with other computers—single team member performs all setup	Apple Configurator required for first-time configuration of each loaner phone

^a^PHI: protected health information.

#### Enterprise Tools for Automated Configuration

Apple’s Device Enrollment Program (DEP) and Volume Purchasing Program (VPP)—unified under Apple Business Manager [9]—together with Mobile Device Management (MDM) software, allow organizations to manage Apple devices that may be redistributed to multiple people over time. Table 2 summarizes the features provided by each enterprise tool.

During initial phone setup, Apple Configurator was used to enroll iPhones into DEP. DEP then contacted the MDM server, and MDM configured the phone remotely, allowing easy automated return processing by any team member, facilitating scalability. The enterprise tools also eliminated the need for Apple IDs on each device, translating into significant time savings. As a result, new iPhones required only 10 minutes and returned iPhones required only 5 minutes to set up. Although we paid a subscription fee for MDM, the efficiency gained greatly outweighed the cost.

**Table 2:** Summary of enterprise tools adopted in iShare 3.0 and key benefits provided by each tool.

Enterprise tool	Key features
Apple Device Enrollment Program	Protect ownership of study team phones and deter theft without using Apple IDs Automatic and mandatory configuration of phones with Mobile Device Management server No cost to use
Apple Volume Purchase Program	Enable bulk automated app download and purchase Download apps without Apple IDs No cost to use
Mobile Device Management	Automate phone configuration (ie, security policies and app installation) Update configuration on previously deployed phones over the Internet Use of *Lost Mode*—enterprise equivalent to *Find My iPhone*—to disable and GPS-track missing phones Monitor availability of iShare inventory Easily reset returned phones with a few clicks Variable subscription fee based on vendor and features desired

#### Cellular Service

We purchased prepaid plans for each phone, which enabled patients without home Wi-Fi to participate in the study; facilitated MDM-initiated resetting of locked returned phones; and allowed activation of Lost Mode on phones not connected to Wi-Fi.

### Study Outcomes

We describe in detail the iShare 3.0 protocol. In addition to the feasibility of the loaner smartphone process, we evaluated process outcomes including the following: number of additional participants enrolled, device return rate, and the estimated per-participant investment cost of a loaner phone. In the final MiCORE analyses, which will be presented in future publications, we will compare iShare participants’ and iPhone owners’ demographics, readmission rates, survey data, and app usage data.

## Results

### Corrie iShare 3.0 Protocol

#### Device Procurement and Preparation

**Figure 1:**
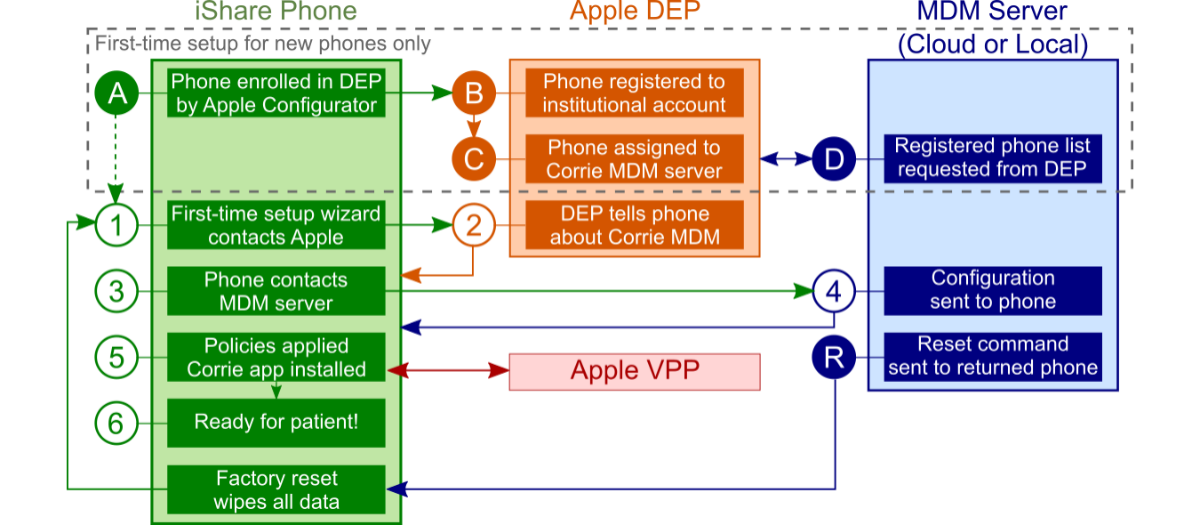
Apple Device Enrollment Program (DEP), Volume Purchasing Program (VPP), and Mobile Device Management (MDM) facilitate automated setup of iShare phones through a series of handoffs. Steps A-D are completed manually the first time an iPhone is adopted into the Corrie iShare program. Steps 1-6 are automatic and require only minimal interaction with the phone. Returned phones are reset with one click through the MDM server (step R), which triggers a factory reset and wipes all data. Phones then automatically proceed through steps 1-6. Physical cleaning and handling steps are not shown in this figure.

Equipment was inventoried and labeled. A SIM card with an active prepaid cellular plan was installed in each phone, and each phone was placed in a protective case. Using Apple Configurator on a Mac computer, each iPhone was updated with the latest version of iOS and provisionally enrolled in Apple DEP and our team’s cloud-based MDM server. DEP and MDM automated the installation and updating of security policies and the Corrie app on each iPhone, while also enabling our team to remotely disable and track lost or stolen phones. Figure 1 depicts a schematic of this process.

Configuration profiles disabled Apple iCloud, disabled Apple ID configuration to prevent participants from enabling Activation Lock on loaner Apple Watches, and blocked bandwidth-heavy streaming apps to limit cellular charges. We did not use MDM to enforce PIN security because the Corrie app already requires it. Apple Watches were paired with each iPhone prior to participant enrollment to ensure they were updated, an unattended process that can take up to several hours.

#### Study Enrollment

Patients were enrolled early in the MiCORE study during their hospitalization for myocardial infarction in accordance with the protocol described previously [10]. Participants with a study-compatible iPhone were assisted with downloading Corrie from the App Store. iPhone owners who did not own an Apple Watch were loaned one, which was paired with their personal device. Participants who did not own a study-compatible iPhone were loaned a team-owned iPhone and prepaired Apple Watch via the Corrie iShare program. Participants began using Corrie while hospitalized and continued to use it at home after hospital discharge. All participants borrowing either an iPhone or Apple Watch were provided with return instructions and a prepaid return package with which to mail equipment back to our team 30 days after hospital discharge. Non-smartphone owners were excluded to remove confounding from the steep learning curve of smartphone adoption.

#### Study Completion and Device Recycling

A total of 30 days after hospital discharge, participants returned borrowed equipment, which was inventoried and disinfected following a hospital-grade protocol. Using MDM, iPhones were factory reset to ensure no PHI remained. DEP and MDM automated the phone configuration (see Figure 1). We then used MDM to update iOS. The Apple Watch was then paired and updated. Equipment was turned off and placed into storage until needed.

### Evaluation of Process Outcomes

We initially purchased 24 iPhone 5S phones on a rolling basis. After protocol refinement and site expansion across three states, we upgraded to 28 iPhone 6S Plus phones. The MiCORE study completed enrollment with a target sample size of 200 patients in April 2019. A total of 92 participants out of 200 (46.0%) were enrolled in the iShare program and 108 (54.0%) participants were enrolled with their personal iPhones. The participants who did not own iPhones would have been excluded from the study without the iShare program. Compared to iPhone owners, iShare participants were slightly younger (age range 30-81 years, mean 57.4 [SD 11] vs age range 32-89 years, mean 60.8 [SD 11]). They were also more likely to be women (33/92, 35.9% vs 25/108, 23.2%), of black race (23/92, 25.0% vs 15/108, 13.9%), and insured by Medicaid (18/92, 19.6% vs 4/108, 3.7%).

As expected, some participants were lost to follow-up, and some devices were lost or damaged. Of participants enrolled with a loaner phone, 72% (66/92) returned the phone and 70% (64/92) returned the watch; however, 2 participants (2%) returned damaged equipment (one phone and one watch), while one package (phone and watch) was damaged by the postal service. Estimating the cost of a refurbished phone at US $250, the loss due to phone nonreturn (n=26) or damage (n=2) was US $7000 or US $76 per iShare participant. The incremental cost of our MDM subscription was US $1950 total for 2 years or US $21 per iShare participant. Thus, we spent US $97 for each iShare participant, resulting in a cost savings of 61% compared to purchasing an iPhone for each participant who did not already own one. This estimated cost savings does not take into account the cost of the smartwatch, which Apple donated to our study team.

Final outcomes of MiCORE and iShare will be presented in a future publication.

## Discussion

In this tutorial, we shared how our team implemented the Corrie iShare program to maximize patient enrollment in the MiCORE study through the use of loaner smartphones and smartwatches.

### Security and Privacy Implications

Data security is paramount for protecting participant privacy. Apple iOS ensures security by encrypting data with the PIN unlock code. We chose to enforce PIN security at the app level rather than by MDM policy; participants could not use Corrie until a PIN had been set on the phone. The advantage of app-based enforcement is that it ensures data security on both iShare phones and personally owned iPhones used in the MiCORE study. While some would advocate for *double enforcement*, we decided to forego an MDM-enforced PIN code, which would have required that our team either not complete the iPhone setup wizard in advance, or set a temporary PIN that would need to be changed at enrollment time. Both situations would prolong study enrollment time without benefit.

To protect participant privacy and ensure that all data were deleted, phones were factory reset after each return. An alternative would have been to clear only user data, which would have reduced turnover time. However, this would have been less complete than a factory reset. While factory reset was initially a laborious and time-consuming process, the automation provided by DEP and MDM mitigated its inconveniences. We also used MDM and VPP to remove the need to preconfigure Apple IDs, eliminating the risk of one participant’s data inadvertently being exposed to a future participant through iCloud data synchronization.

### Institutional Information Technology Challenges

#### Overview

As with many novel protocols in large institutions, our initial iShare protocol encountered a number of challenges. While our health system had a process for mobile devices that would be used by patients sequentially while remaining hospitalized, it did not offer guidance for implementing our desired process where a patient uses a loaner phone while hospitalized and continues to use it at home for a time before returning it. We share the lessons we learned to help other mHealth researchers implement a loaner device program.

#### Apple Enterprise Tools

Apple DEP and VPP are offered to institutions and corporations rather than independent teams within those entities. As a result, it was necessary to identify and collaborate with the information technology (IT) staff member responsible for overseeing DEP and VPP. However, these Apple services are also available to any business at no cost and are not limited to large institutions, making this a valuable resource to independent teams, which could register as small businesses.

#### Mobile Device Management and Wi-Fi

We launched iShare using a cloud-based MDM solution provided by Jamf Software, LLC. Subsequently, our IT department offered us free access to use the institutional enterprise MDM—VMware AirWatch—which would have saved us the cost of subscribing to Jamf and enabled access to the hospital-authenticated Wi-Fi network. However, unlike Jamf, our institutional MDM required access to our institution’s network to manage the phones and thus would not scale to a multi-site study. The interface was also more difficult for nontechnical users to learn. Therefore, we continued to use our team’s independent MDM without authenticated Wi-Fi. Since iOS in standby does not stay connected to guest Wi-Fi networks, participants initially struggled to use Corrie with hospital Wi-Fi. This issue was mitigated after we added cellular data service to iShare, but increased cellular data expenses in an environment that otherwise had Wi-Fi.

### Loaner Retrieval

We initially asked that participants return loaner equipment 30 days after discharge. Initially, only basic instructions were given to patients on how to return equipment. Our loaner return system evolved to include written instructions, prepaid mailers, and follow-up reminder calls.

We discovered that reminder phone calls made from personal phones were less likely to be answered or returned. Therefore, we switched to using hospital-based phones, which patients were more receptive to answering. Although we did not have a dedicated phone number or voicemail, either could be useful in the future to facilitate receiving callbacks from patients.

The final step in retrieving unreturned devices was using *Lost Mode* GPS tracking. The vast majority of unreturned phones could not be GPS-tracked because the phone batteries had died. MDM data confirmed these phones had not connected to the Internet for a period of time and were presumed lost. DEP mitigated the risk of theft as participants could not bypass our security restrictions even if phones were reset. Our return rate was higher than one might anticipate in the absence of a return incentive.

Potential methods of increasing the return rate include actively monitoring MDM data and contacting patients if phones are unused for 7 days, rather than waiting until the end of the 30-day period to contact participants who had not returned their phones. Automated email and text reminders to patients or a monetary return incentive would also likely increase returns.

### Apple Watch Limitations

Apple Watch setup, although simple, can take several hours to install updates and cannot be automated using Apple Configurator or MDM because of current software limitations, which limits scalability. In addition, a participant could accidentally *take ownership* of an Apple Watch by configuring their Apple ID on the phone, which enables Activation Lock on the watch. While DEP can bypass Activation Lock on iPhones, no bypass is available for Apple Watches; thus, we used MDM to disable Apple ID configuration. However, this reduced the functionality of loaner phones by making some apps and features unavailable.

### Conclusions

The Corrie iShare program demonstrates the potential for sustainable and scalable smart technology reuse. A loaner smartphone program enables teams to focus on app development while maximizing study enrollment. Smartphone enterprise features readily adapt to a loaner program that ensures security and privacy. Although the loaner process described here is limited to iOS devices, the overall framework may be generalizable to other platforms. Implementation on other platforms would be an area for future research. In addition, while our work is promising for expanding demographic reach, we excluded patients who did not own a smartphone; thus, further research is necessary to study the feasibility of using a loaner program to reach the 39% of the global population who do not own smartphones [11] and are not typically represented in mHealth research, yet may stand to benefit from these technologies.

## Appendix

Multimedia Appendix 1 Final protocol in step-by-step format.

## Abbreviations

DEP: Device Enrollment Program
IT: information technology
MDM: Mobile Device Management
MiCORE: Myocardial infarction COmbined-device Recovery Enhancement
NIH: National Institutes of Health
NINR: National Institute of Nursing Research
NRSA: National Research Service Award
PHI: protected health information
VPP: Volume Purchasing Program
