# Correction: Strategies for the Successful Implementation of a Novel iPhone Loaner System (iShare) in mHealth Interventions: Prospective Study

**DOI:** 10.2196/31472

**Published:** 2021-09-20

**Authors:** William E Yang, Erin M Spaulding, David Lumelsky, George Hung, Pauline Phuong Huynh, Kellen Knowles, Francoise A Marvel, Valerie Vilarino, Jane Wang, Lochan M Shah, Helen Xun, Rongzi Shan, Shannon Wongvibulsin, Seth S Martin

**Affiliations:** 1 School of Medicine Johns Hopkins University Baltimore, MD United States; 2 School of Nursing Johns Hopkins University Baltimore, MD United States; 3 Krieger School of Arts and Sciences Johns Hopkins University Baltimore, MD United States; 4 Ciccarone Center for the Prevention of Cardiovascular Disease, Division of Cardiology School of Medicine Johns Hopkins University Baltimore, MD United States; 5 Department of Biomedical Engineering School of Medicine Johns Hopkins University Baltimore, MD United States

In “Strategies for the Successful Implementation of a Novel iPhone Loaner System (iShare) in mHealth Interventions: Prospective Study” (JMIR Mhealth Uhealth 2019;7(12):e16391) the authors noted two errors.

In the *Results* section of the originally published paper, the first paragraph of the subsection “Evaluation of Process Outcomes” included incorrect numerators and denominators in the statistical values introduced during the copyediting process. The final two sentences of this paragraph were originally published as follows:

Compared to iPhone owners, iShare participants were slightly younger (age range 30-81 years, mean 57.4 [SD 11] vs age range 32-89 years, mean 60.8 [SD 11]). They were also more likely to be women (72/200, 36.0% vs 45/200, 23.0%), of black race (50/200, 25.0% vs 28/200, 14.0%), and insured by Medicaid (40/200, 20.0% vs 8/200, 4.0%).

This has been corrected to:

Compared to iPhone owners, iShare participants were slightly younger (age range 30-81 years, mean 57.4 [SD 11] vs age range 32-89 years, mean 60.8 [SD 11]). They were also more likely to be women (33/92, 35.9% vs 25/108, 23.2%), of black race (23/92, 25.0% vs 15/108, 13.9%), and insured by Medicaid (18/92, 19.6% vs 4/108, 3.7%).

As well, the phone number of the Corresponding Author has been updated from the originally published number to “1 410 550 0100.”

The correction will appear in the online version of the paper on the JMIR Publications website on September 20, 2021, together with the publication of this correction notice. Because this was made after submission to PubMed, PubMed Central, and other full-text repositories, the corrected article has also been resubmitted to those repositories.

