# Supplementary material for: Strategies for the Successful Implementation of a Novel iPhone Loaner System (iShare) in mHealth Interventions: Prospective Study
Source: JMIR Mhealth Uhealth. 2019 Dec 16;7(12):e16391. doi: 10.2196/16391 (PMC6937543; doi:10.2196/16391)
Supplement: Multimedia Appendix 1 [file mhealth_v7i12e16391_app1.docx]

**iShare Preparation Guide**

NOTE: This guide is NOT for bedside deployment. These instructions are on how to prepare an iPhone and Apple Watch for iShare deployment, and are completed well in advance of the bedside visit.

Newly Purchased Phone: Initial setup

1. Charge Phone and Watch fully
2. Insert SIM
3. Connect Phone to deployment computer and open Apple Configurator (AC)
4. AC: Right click phone and "Update..." phone OS
5. Claim iShare ID number from inventory spreadsheet (type starting with apostrophe so Excel doesn't try to format it, I.e. apostrophe zero zero one two)
   1. Enter serial number (**Phone AND Watch**), SIM #, SIM PUK in spreadsheet
   2. Put custom label on back of phone, which should include the iShare ID
   3. Put phone into protective case
   4. Remember to also tag/label iPhone charge plug, iPhone charge cord, Watch charge plug, Watch charge cord.
6. Go to “DEP Enrollment”

DEP Enrollment

1. AC: Right click phone and Apply -> iShare DEP Blueprint
   1. Once phone starts to reset, insert SIM, then add SIM # and PUK to inventory spreadsheet
      1. FYI: Inserting SIM before starting OS restore (if PIN locked) rarely cause errors but usually works fine
   2. AC will ask for the DEP/VPP account ([user ID redacted]) password
2. Go to <http://deploy.apple.com/>
   1. Log in with [user ID redacted]
   2. Go to “Device Enrollment Program” -> “Manage Devices”
   3. Paste the serial number of the phone(s) and assign to your Mobile Device Management server
3. In JAMF: [URL redacted]
   1. Mobile Devices -> PreStage Enrollments -> iShare Prestage -> Refresh
   2. On the phone, proceed through iOS setup (enter your user ID and enrollment keyword when prompted)
   3. Back to JAMF: find the mobile device by serial number
   4. In the General section, click “Edit”
   5. Check “Enforce Mobile Device Name”
   6. Set “Mobile Device Name” to "[Site of deployment] Phone xxxx" (iShare ID from above)
   7. Set “Asset Tag” to the 4 digit iShare ID
   8. Save
   9. If needed, go to Management tab -> “Send Blank Push” to clear any pending commands
4. **Skip to “Watch Setup” below.**

Recycle and Refresh

1. Charge Phone and Watch fully
2. In JAMF: [URL redacted]
   1. Click Mobile Devices and search for the 4 digit iShare ID
   2. Click the correct phone
   3. Go to the Management tab
   4. Click the Wipe Device icon
      1. If present, check “Clear Activation Lock”
      2. Confirm “Wipe Device”
3. If the Phone does not start to reset itself
   1. Make sure it is connected to Wi-Fi
   2. If it is PIN locked, you’ll need to manually use iTunes to do a Restore
      1. Press and hold Power+Home until iTunes restore screen shows up
      2. Connect iPhone to computer (USB cable) and start iTunes
      3. Follow the prompts to do a Restore
4. Click through iOS setup
   1. When prompted, log in with your user ID and your deployment keyword
5. Update iOS
6. Unpairing a Watch factory resets it. If a returned Watch is still paired:
   1. Press and hold the side button until you see Power Off
   2. Firmly press Power Off for a moment
   3. Tap “Erase all content and settings” to reset the Watch
7. Continue to Watch Setup below

Watch Setup

1. Pair the Watch (this could take hours if it needs to update)
2. **Watch app -> General -> About**
   1. **Name: "[Site of deployment] Watch xxxx"**
3. Also, update watchOS if it didn’t automatically when you first paired
